# Injectable Excipients as Novel Influenza Vaccine Adjuvants

**DOI:** 10.3389/fmicb.2019.00019

**Published:** 2019-01-24

**Authors:** Huapeng Feng, Makoto Yamashita, Tiago Jose da Silva Lopes, Tokiko Watanabe, Yoshihiro Kawaoka

**Affiliations:** ^1^Division of Virology, Department of Microbiology and Immunology, Institute of Medical Science, University of Tokyo, Tokyo, Japan; ^2^Department of Pathobiological Sciences, School of Veterinary Medicine, University of Wisconsin–Madison, Madison, WI, United States; ^3^Department of Special Pathogens, International Research Center for Infectious Diseases, Institute of Medical Science, University of Tokyo, Tokyo, Japan

**Keywords:** injectable excipients, influenza, vaccine, adjuvants for vaccines, protective efficacy

## Abstract

Influenza outbreaks can be either seasonal or pandemic. Vaccination is an effective strategy to control influenza; however, the efficacy of the currently available inactivated influenza virus vaccines is suboptimal, especially in the elderly. Vaccine efficacy can be improved by the addition of adjuvants, but few adjuvants have been approved for human vaccines. To explore novel, safe, and effective adjuvants for influenza vaccines, here we used a mouse model to screen 46 injectable drug additives approved in Japan. Of these 46 candidates, we identified 20 compounds that enhanced the efficacy of the split influenza HA vaccine against lethal virus challenge. These 20 compounds included 15 novel adjuvant candidates and 5 compounds with previously reported adjuvant effects for other antigens but not for influenza vaccine. Given that these additives are already approved for human use, the hurdle for their clinical use as novel and effective adjuvants for influenza or other vaccines is lower than for other adjuvant candidates whose safety profiles are unknown.

## Introduction

Influenza viruses cause annual epidemics that peak during the winter, and occasional pandemics that result in considerable morbidity and mortality worldwide. Annual vaccination is considered to be one of the most effective ways to prevent seasonal influenza. Current seasonal vaccines are composed of three or four different influenza virus strains (H1N1, H3N2, and one or two influenza B viruses), which are reviewed annually and updated to match the antigenicity of the circulating strains; however, the overall efficacy of current seasonal influenza vaccines is suboptimal, especially for H3N2 viruses, at around 30% or less, even when the vaccine strain matches the circulating virus (Dominguez et al., 2016; Flannery et al., 2018)^1^ and is much lower if the vaccine strain and the dominant circulating influenza virus are mismatched (Chambers et al., 2015; Zimmerman et al., 2016).

The immunogenicity of influenza vaccines (i.e., their ability to induce humoral and/or cell-mediated immune responses) can be improved by the addition of adjuvants (Tetsutani and Ishii, 2012). Aluminum salt, which is referred to as ‘alum,’ has been the most widely used adjuvant in human vaccines since its discovery in the 1920s, and squalene-based oil-in-water emulsions (e.g., MF59 and AS03) have recently been licensed as adjuvants for human influenza vaccines in Canada, Europe, and Latin America (Weir and Gruber, 2016; Wilkins et al., 2017). However, the efficacy of some adjuvanted influenza vaccines remains suboptimal (Manzoli et al., 2011) and concerns have been raised regarding their safety, most recently after reports of an increased incidence of narcolepsy in children that received a squalene-adjuvanted pandemic influenza vaccine (Nohynek et al., 2012; Szakacs et al., 2013). Squalene adjuvants also cause local and systemic reactogenicity (Fox and Haensler, 2013; Petrovsky, 2015). Therefore, safe and non-reactogenic influenza vaccine adjuvants that can elicit strong protective immunity are urgently needed.

Substances already approved for human use with good safety profiles are ideal candidates to evaluate as potential adjuvants. Excipients are natural or synthetic additives that serve as the vehicle or medium for a drug or other active ingredient, mainly to enhance the action of the active ingredient or promote its dissolution and absorption. Excipients are also used in manufacturing to aid in the stability of active substances and extend their shelf lives. Split influenza vaccines, one of three types of inactivated influenza vaccines (i.e., whole-virus vaccines, split-virus vaccines, and subunit vaccines), are usually administered intramuscularly or subcutaneously, and approved injectable excipients with strong safety profiles can be injected into humans. One excipient, hydroxypropyl-beta-cyclodextrin, showed adjuvanticity for the influenza split vaccine via the MyD88- and TBK1-dependent pathways upon subcutaneous administration to C57BL/6J mice and cynomolgus macaques (Onishi et al., 2015). This compound was shown to act as a potent mucosal adjuvant for seasonal and pandemic influenza vaccines against sub-heterologous virus infection (Kusakabe et al., 2016). Therefore, selection from injectable excipients is an effective approach to identify novel adjuvants with good safety profiles. To explore adjuvant candidates that are safe and exhibit strong protective immunity against influenza virus infection, here we screened 46 injectable excipients for adjuvant effects with the current seasonal influenza vaccine in mice. We identified 20 compounds that enhanced influenza virus-specific antibody responses and the efficacy of an influenza HA vaccine against a lethal challenge of influenza virus. Our findings will facilitate the development of novel, effective adjuvanted human influenza vaccines and vaccines against other infectious and non-infectious diseases.

## Materials and Methods

### Cells and Viruses

Madin-Darby canine kidney (MDCK) cells were maintained in minimum essential medium (MEM) (Gibco) supplemented with 5% newborn calf serum (Sigma) at 37°C in 5% CO_2_. MDCK cells were used for plaque assays to titrate viruses.

Mouse-adapted A/California/04/2009 virus (H1N1; MA-CA04) generated in our laboratory as previously described (Sakabe et al., 2011) was used to challenge mice. The stock titer of MA-CA04 was 2.0 × 10^7^ plaque forming unit (PFU)/ml. A/California/07/2009 virus (H1N1; CA07), which was isolated early in the 2009 pandemic and is one of the components of the split influenza HA vaccine, was used as an antigen for the ELISA to determine the virus-specific antibody titers of sera obtained from the immunized mice. There are four amino acid differences between the HA protein of CA07 and that of MA-CA04 (i.e., HA-D127E, HA-K142N, HA-A197T, and HA-D222G).

### Influenza Vaccines and Compounds Used for the Screen

Quadrivalent split influenza HA vaccines were obtained from DENKA SEIKEN, Co., Ltd. (Japan). The quadrivalent split influenza HA vaccine (for the 2016–2017 influenza season), which contains the HA proteins of CA07 (H1N1), A/Hong Kong/4801/2014 (H3N2), B/Phuket/3073/2013 (Yamagata lineage), and B/Texas/2/2013 (Victoria lineage), was used for the screen and to test virus replication in immunized mice after virus challenge, except for when we tested ethanol, which was one of the compounds used for the screen. Ethanol was tested with the quadrivalent split influenza HA vaccine for the 2015–2016 influenza season, which contains the HA proteins of CA07 (H1N1), A/Switzerland/9715293/2013(H3N2), B/Phuket/3073/2013 (Yamagata lineage), and B/Texas/2/2013 (Victoria lineage). For compound testing, aluminum hydroxide gel Alhydrogel^®^ adjuvant 2% (alum), purchased from InvivoGen, was used as a positive control [antigen:alum (v/v) = 1:1] (approximately equal to 500 μg alum/dose). All other compounds tested were injectable excipients purchased from the companies listed in Supplementary Table S1. These compounds were suspended in phosphate-buffered saline (PBS; without calcium or magnesium) at concentrations of 10 mg/ml or 10 μl/ml and were sonicated in a water bath for 15 min at room temperature. The compound stocks were then stored at -20°C until use except for alum, which was stored at room temperature. Before being mixed with the split influenza HA vaccine, the suspensions or solutions were thawed and sonicated again for 5 min.

### Immunization and Protection

Five-week-old female BALB/c mice were purchased from Japan SLC, Inc. After 1 week of adaptation, the mice were immunized with a suboptimal dose of influenza HA vaccine [0.001 μg/dose (2016–2017 season) calculated on the basis of the amount of HA from CA07 (H1N1) [for ethanol, 0.003 μg/dose (2015–2016 season)] with or without compounds via intramuscular injection into the gastrocnemius muscle (four mice per group). Two weeks later, the mice were boost immunized intramuscularly. On day 14 after the boost immunization, blood was collected via the facial vein by using a goldenrod animal lancet (5 mm), and sera were isolated for measuring virus-specific antibody titers. Three weeks after the boost immunization, the immunized mice were challenged intranasally, under anesthesia, with 10 MLD_50_ (dose required to kill 50% of infected mice; which was equivalent to 6.8 × 10^5^ PFU/50 μl/mouse) of MA-CA04 virus. Body weight and survival was monitored daily for 14 days after virus challenge. Mice that lost more than 25% of their original body weight were euthanized. To determine virus titers in mice, organs were harvested on days 3 and 6 post-challenge and homogenized and titrated on MDCK cells by using a plaque assay as described previously (Gaush and Smith, 1968).

### Measurement of Virus-Specific Antibody Titers

The virus-specific antibody titers in the sera were determined by using a modified ELISA as previously described (Uraki et al., 2014). Briefly, 96-well ELISA plates (IWAKI) were coated with 6 μg/ml of inactivated and purified CA07 virus solution overnight at 4°C (50 μl/well). The plates were then blocked with 200 μl of 20% Blocking One (Nacalai) in water at room temperature for 1 h. After blocking, the plates were washed once with PBS containing 0.05% Tween-20 (PBS-T), and then twofold serially diluted serum samples were added to the plates, followed by a 1-h incubation at room temperature. Bound IgG was detected by using peroxidase-labeled goat anti-mouse IgG (gamma) antibody, F (ab′) 2 fragment (Kirkegaard & Perry Laboratory, Inc.). After the plates were washed four times with PBS-T, 100 μl of 2, 2′-azino-*bis* (3-ethylbenzothiazoline-6-sulfonic acid) diammonium salt substrate solution was added to each well to initiate the color reaction, and the OD was measured at a wavelength of 405 nm. The antibody titer was defined as the reciprocal of the highest serum dilution that produced an OD_405_ > 0.1 after correcting for the negative serum control (Even-Or et al., 2010).

### Statistical Analysis

We used R^2^ and lme4 (Bates et al., 2015) to perform a linear mixed effects analysis of the body weight data, which were normalized to the initial weight of each individual animal. As fixed effects, we used the different treatment groups (i.e., vaccine alone, vaccine plus compound, and vaccine plus alum), and the time of the measurement (with an interaction term between those fixed effects). As random effects, we had intercepts for the individual animals. We used the lsmeans (Lenth, 2016) package to compare the groups at different time points, for each model separately, and the *P*-values were adjusted using Holm’s method. For comparisons of virus titers, we used two-sided unpaired *t*-tests, between the different treatment groups (i.e., vaccine alone, vaccine plus compound, and vaccine plus alum). For the analysis of the survival data, we used the Log-rank test, comparing the vaccine plus compounds or alum to the vaccine alone group. We used the OASIS 2 (Han et al., 2016) software for this analysis. *P*-values of < 0.05 were considered statistically significant.

### Ethics Statement

All experiments with mice were performed in the biosafety level 2 containment laboratory in the Institute of Medical Science, the University of Tokyo (Tokyo, Japan) in accordance with the Regulations for Animal Care of the University of Tokyo and the Guidelines for Proper Conduct of Animal Experiments by the Science Council of Japan and were approved by the Animal Experiment Committee of the Institute of Medical Science, the University of Tokyo (Approval No. PA14-38).

## Results and Discussion

### Identification of 21 Compounds That Enhance the Humoral Responses to an Influenza Vaccine in Mice

To explore novel adjuvants for inactivated influenza vaccine, we conducted a screen in a mouse model of 46 injectable excipients, which were selected from a library of injectable compounds approved for i.m. or s.c. administration in Japan. Commercially available alum adjuvant was used as a positive control, as described in Section “Materials and Methods,” because alum is the most globally and historically used adjuvant, having been used in many clinical studies (Tetsutani and Ishii, 2012). First, we performed an optimization experiment to determine the optimum dose of HA vaccine for our screen. We found that a dose of 0.001 μg of HA vaccine elicited virus-specific antibodies only when the alum adjuvant was administered to mice together with the vaccine, whereas a dose of 0.003 μg of HA vaccine induced virus-specific antibodies both in the presence and absence of alum (Supplementary Figure S1), indicating that a dose of 0.001 μg of HA vaccine was appropriate to use to immunize mice in our screen.

**Figure 1:**
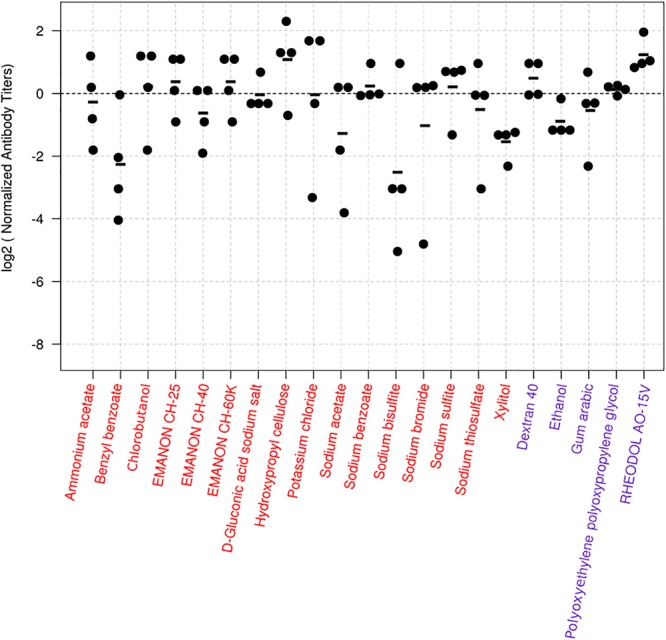
Virus-specific antibody titers induced in mice by the 24 hit compounds in combination with HA vaccine. Six-week-old BALB/c mice were immunized with influenza HA vaccine with or without compounds twice with a 2-week interval between the vaccinations. Blood samples were collected 2 weeks after the second immunization. Virus-specific antibodies were measured by using an ELISA with inactivated and purified CA07 virus as the coating antigen. Depicted are the antibody titers obtained from the mice immunized with the vaccine plus the candidate compounds. Each dot represents one mouse; the individual antibody titers were divided by the average antibody titer of the mice immunized with the vaccine plus alum in the same batch. This procedure normalizes the values from the animals immunized with candidate compounds to their respective controls, and the log transformation helps with the interpretation of the values. Values above zero indicate that the antibody titers of the mice treated with the vaccine plus the candidate compound were higher than those of their controls. The black horizontal line represents the mean antibody titers from individual mice (*n* = 4). The dotted line represents the reference vaccine plus alum. Compounds depicted in red are totally novel adjuvant candidates, whereas those in blue are novel adjuvant candidates for influenza vaccine.

For the screen, mice were immunized twice with PBS, compounds alone (100 μg/dose), HA vaccine only, or HA vaccine plus compounds [100 μg/dose; except for ethanol which was used at 10% (v/v)] by intramuscular administration in a 100-μl volume with a 2-week interval between the vaccinations. HA vaccine plus commercial alum was used as a positive control. Two weeks after the boost immunization, serum samples were obtained from the immunized mice and examined for the presence of virus-specific antibodies by use of an ELISA. No antibodies against CA07 virus were detected in the PBS and compound alone groups. Most of the mice immunized with the HA vaccine alone produced no virus-specific antibodies (Table 1 and Supplementary Table S1), but a few mice in this group did exhibit low levels of virus-specific antibodies (i.e., antibody titers of 1:10 or 1:20) (Supplementary Table S1). We defined hits as compounds that induce higher levels of mean antibody titers when combined with the HA vaccine compared with the HA vaccine alone; three compounds were excluded because the antibody titers in some of the mice exposed to these compounds were ≤1:20, the titers found in the HA vaccine alone group (Supplementary Table S1). Based on these selection criteria, we selected 21 compounds that enhanced antibody production compared with HA vaccine alone (Table 1). These 21 hits included 16 novel adjuvant candidates and 5 novel adjuvant candidates for influenza vaccine; that is, their adjuvant activity had been reported for other antigens but not for influenza vaccine. Hydroxypropyl cellulose induced the highest level of virus-specific antibody production (higher than that of alum) (Figure 1, Table 1 and Supplementary Table S1).

**Table 1:** Virus-specific antibody titers in sera from immunized mice for the 21 hit compounds identified in the screen^a^.

	Mean of Ab. titer^b^				
	Compound	Vaccine	Vaccine +	Vaccine +	Ab. titer ratio
Compound	only	alone	compound	alum	(compound/alum)^c^	Status^d^
Ammonium acetate	<10	<10	1200.00	1120.00	1.07	a
Benzyl benzoate	<10	<10	460	1320.00	0.35	a
Chlorobutanol	<10	<10	1680.00	1120.00	1.50	a
Dextran 40	<10	10, 20^e^	1920.00	1320.00	1.45	b
EMANON CH-25	<10	<10	3520.00	2400.00	1.47	a
EMANON CH-40	<10	<10	1760.00	2400.00	0.73	a
EMANON CH-60K	<10	<10	3520.00	2400.00	1.47	a
Ethanol	<10	<10	400.00	720.00	0.56	b
D-Gluconic acid sodium salt	<10	<10	1600.00	1600.00	1.00	a
Gum arabic	<10	<10	1360.00	1600.00	0.85	b
Hydroxypropyl cellulose	<10	<10	5440.00	2080.00	2.62	a
Polyoxyethylene polyoxypropylene glycol	<10	<10	2560.00	2400.00	1.07	b
Potassium chloride	<10	<10	2920.00	1600.00	1.83	a
RHEODOL AO-15V	<10	10, 20	3200.00	1320.00	2.42	b
Sodium acetate	<10	<10	740.00	1120.00	0.66	a
Sodium benzoate	<10	<10	1600.00	1320.00	1.21	a
Sodium bisulfite	<10	<10	730.00	1320.00	0.55	a
Sodium bromide	<10	<10	970.00	1120.00	0.87	a
Sodium sulfite	<10	<10	2080.00	1600.00	1.30	a
Sodium thiosulfate	<10	10, 20	1320.00	1320.00	1.00	a
Xylitol	<10	<10	560.00	1600.00	0.35	a

^a^*Six-week-old BALB/c mice were intramuscularly immunized with the indicated immunogens (100 μl) twice with a 2-week interval between immunizations. Four mice were used per group. The serum samples were collected 2 weeks after the second immunization to measure virus-specific antibody titers.*

^b^*The virus-specific antibody titers (Ab. titers) were determined by use of an ELISA with inactivated CA07 virus as the coating antigen. The OD was measured at a wavelength of 405 nm. The antibody titer was defined as the reciprocal of the highest serum dilution that produced an OD_405_> 0.1 after correcting for the negative serum control. The values are the means of the four individual antibody titers per group.*

^c^*The Ab. titer ratio = [mean of Ab. titer (vaccine + compound)]/[mean of Ab. titer (vaccine + alum)].*

^d^*(a) Novel adjuvants; (b) Novel adjuvants for influenza vaccine, that is, their adjuvanticity has been reported for other antigens but not for influenza vaccine.*

^e^*Among four mice tested, the antibody titers in two animals were 20 and 10, whereas those in the other two were < 10*.

### Identification of 20 Compounds That Enhance the Protective Efficacy of Influenza Vaccine Against Lethal Virus Challenge in Mice

To further explore whether these compounds could enhance the protective efficacy of influenza vaccine, the immunized mice were challenged with 10 MLD_50_ of MA-CA04 virus 1 week after blood collection (3 weeks after the boost immunization), and body weight and survival were monitored for 14 days. Among these 21 compounds, benzyl benzoate did not increase survival after challenge compared with the HA vaccine only group (Supplementary Table S1), and this compound was therefore excluded from further assessment. We thus identified 20 compounds whose protective efficacy was similar or superior to that of alum on the basis of survival rates (Table 2). These 20 hit compounds included 15 novel adjuvant candidates and 5 novel adjuvant candidates for influenza vaccine (Tables 1, 2).

**Table 2:** Protective efficacy of the 20 hit compounds against lethal challenge of immunized mice^a^.

									Enhanced	
									protective efficacy	
Compound	Maximum body weight loss% ± SD^b^				Protective efficacy (survival/total)				(%) compared to:^c^	
	Compound	Vaccine	Vaccine +	Vaccine +	Compound	Vaccine	Vaccine +	Vaccine +	Vaccine	Vaccine +
	only	alone	compound	alum	only	alone	compound	alum	alone	alum
Ammonium acetate^∗^	24.9	24.7	22.7 ± 0.3	18.9 ± 8.4	0/4	0/4	2/4	2/4	50	0
Chlorobutanol^∗^	24.7 ± 2.0	22.3	21.3 ± 2.5	18.9 ± 8.4	0/4	0/4	3/4	2/4	75	25
Dextran 40	23.1 ± 0.6	24.7	22.6 ± 1.1	18.0 ± 3.7	0/4	0/4	3/4	3/4	75	0
EMANON CH-25^∗^	23.3 ± 1.5	23.3 ± 0.8	19.1 ± 1.9	20.0 ± 4.3	0/4	0/4	4/4	2/4	100	50
EMANON CH-40^∗^	24.0 ± 0.9	23.3 ± 0.8	20.4 ± 4.0	20.0 ± 4.3	0/4	0/4	3/4	2/4	75	25
EMANON CH-60K^∗^	23.7	23.3 ± 0.8	19.5 ± 3.3	20.0 ± 4.3	0/4	0/4	4/4	2/4	100	50
Ethanol	23.1 ± 1.0	22.4 ± 0.7	22.6 ± 2.0	20.9 ± 2.4	0/4	0/4	3/4	3/4	75	0
D-Gluconic acid sodium salt^∗^	23.60 ± 1.8	23.0	21.8 ± 1.1	20.8 ± 1.0	0/4	0/4	3/4	2/4	75	25
Gum arabic	21.1 ± 0.6	24.3	18.9 ± 4.3	20.0 ± 4.2	0/4	0/4	3/4	2/4	75	25
Hydroxypropyl cellulose^∗^	21.6 ± 0.5	25.0	18.9 ± 2.9	24.2 ± 0.8	0/4	0/4	4/4	2/4	100	50
Polyoxyethylene polyoxypropylene glycol	24.6	23.3 ± 0.8	20.5 ± 0.9	20.0 ± 4.3	0/4	0/4	4/4	2/4	100	50
Potassium chloride^∗^	25.0	23.0	19.0 ± 5.3	20.8 ± 1.0	0/4	0/4	2/4	2/4	50	0
RHEODOL AO-15V	23.4 ± 1.1	24.7	21.3 ± 5.1	18.0 ± 3.7	0/4	0/4	3/4	3/4	75	0
Sodium acetate^∗^	23.0 ± 1.5	24.7	23.1 ± 2.0	18.9 ± 8.4	0/4	0/4	2/4	2/4	50	0
Sodium benzoate^∗^	24.6	21.6 ± 2.5	20.8 ± 2.9	17.7 ± 3.9	0/4	0/4	4/4	2/4	100	50
Sodium bisulfite^∗^	22.5 ± 0.5	21.6 ± 2.5	22.2 ± 0.6	17.7 ± 3.9	0/4	0/4	3/4	2/4	75	25
Sodium bromide^∗^	21.3 ± 1.6	24.7	21.0 ± 2.0	18.9 ± 8.4	0/4	0/4	3/4	2/4	75	25
Sodium sulfite^∗^	23.3 ± 0.7	23.0	20.9 ± 3.6	20.8 ± 1.0	0/4	0/4	4/4	2/4	100	50
Sodium thiosulfate^∗^	20.4	24.7	20.9 ± 2.0	18.0 ± 3.7	0/4	0/4	3/4	3/4	75	0
Xylitol^∗^	23.0 ± 0.7	24.0	23.2 ± 1.4	20.8 ± 1.0	0/4	0/4	2/4	2/4	50	0

^a^*Six-week-old BALB/c mice were intramuscularly immunized with the indicated immunogens (100 μl) twice with a 2-week interval between immunizations. Four mice were used per group. Three weeks after the second immunization, the mice were challenged with 10 MLD_50_ of MA-CA04 virus. Body weight and survival were monitored every day for 14 days.*

^b^*Maximum percentage weight loss (mean ± SD) is shown.*

^c^*Enhanced protective efficacy refers to the survival rate for the HA vaccine plus compound group compared to the HA vaccine alone group or the HA vaccine plus alum group.*

^∗^*Novel adjuvant candidates*.

**Figure 2:**
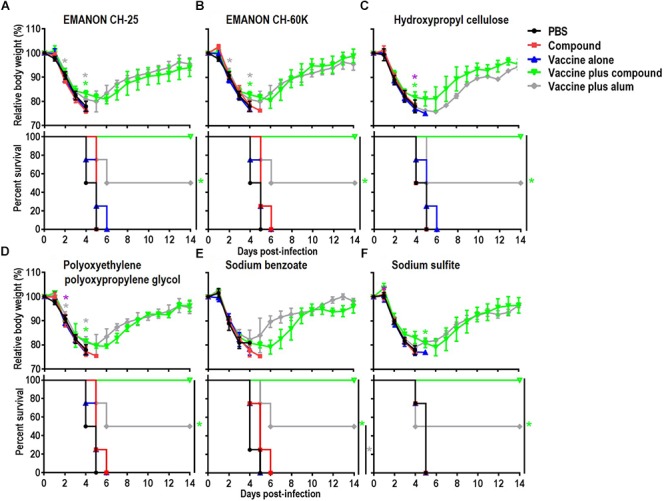
Body weight changes and survival rates of immunized mice after lethal challenge. Six-week-old BALB/c mice were mock-immunized with PBS or compounds only, or they were immunized with HA vaccine alone or compound-adjuvanted HA vaccine twice with a 2-week interval between vaccinations. Mice were intranasally challenged with 10 MLD_50_ of MA-CA04 virus 3 weeks after the second immunization. Body weight and survival were monitored for 14 days. The body weight data shown are means and standard deviation (SD) (*n* = 4). **(A)** EMANON CH-25; **(B)** EMANON CH-60K; **(C)** Hydroxypropyl cellulose; **(D)** Polyoxyethylene polyoxypropylene glycol (160E.O.) (30P.O.); **(E)** Sodium benzoate; **(F)** Sodium sulfite. Green asterisks indicate a significant difference between the vaccine alone and the vaccine plus compound groups; gray asterisks indicate a significant difference between the vaccine alone and the vaccine plus alum groups; purple asterisks indicate a significant difference between the vaccine plus alum and the vaccine plus compound groups. ^∗^*P* < 0.05.

Among the 15 novel hits, 5 compounds (i.e., EMANON CH-25, EMANON CH-60K, hydroxypropyl cellulose, sodium benzoate, and sodium sulfite) completely protected mice when immunized with HA vaccine from lethal challenge with MA-CA04 virus (Figure 2 and Table 2). All of these five compounds induced equal or higher mean titers of virus-specific antibodies relative to that of alum, although no significant difference was detected (Figure 1 and Table 1). Six of the 15 compounds (i.e., Chlorobutanol, EMANON CH-40, D-gluconic acid sodium salt, sodium bisulfite, sodium bromide, and sodium thiosulfate) when administered with the vaccine provided 75% protective efficacy against lethal challenge (Table 2 and Supplementary Figure S2). In addition, four other compounds (i.e., ammonium acetate, potassium chloride, sodium acetate, and xylitol) protected 50% of immunized mice from lethal infection when combined with the HA vaccine (Table 2 and Supplementary Figure S2). These compounds induced lower or comparable antibody titers to alum in the immunized mice, but their protective efficacy was comparable to that of alum (Figure 1 and Table 1).

Five of our hit compounds have shown adjuvant effects for other antigens, but their adjuvanticity for influenza vaccine had not been reported [i.e., dextran 40, ethanol, gum arabic, polyoxyethylene polyoxypropylene glycol (160E.O.) (30P.O.), and RHEODO L AO-15V]. Among them, polyoxyethylene polyoxypropylene glycol (160E.O.) (30P.O.) induced a slightly higher antibody titer than that induced by alum and provided complete protection from lethal infection; that is, all of mice immunized with this compound plus HA vaccine survived, whereas two of the four mice that received alum plus HA vaccine died after lethal challenge (Figure 2 and Tables 1, 2). The HA vaccine in combination with dextran 40, ethanol, gum arabic, or RHEODOL AO-15V provided 75% protection against lethal challenge (Table 2 and Supplementary Figure S2).

### Effect of Immunization of Mice With HA Vaccine Together With the Top Six Compounds on the Replication of Challenge Virus in the Respiratory Tract

To examine the effects of promising adjuvant candidates on virus replication in immunized mice after challenge, we selected 6 of the 20 positive compounds [EMANON CH-25, EMANON CH-60K, hydroxypropyl cellulose, polyoxyethylene polyoxypropylene glycol (160E.O.) (30P.O.), sodium benzoate, and sodium sulfite] that were identified as novel adjuvant candidates for influenza vaccine, induced comparable or higher antibody titers compared to alum, and showed complete protective efficacy (Figure 2 and Tables 1, 2), for further testing. Six mice per group immunized with HA vaccine and the respective compound were challenged 3 weeks after the second immunization with 10 MLD_50_ of MA-CA04 virus, and organ samples [i.e., nasal turbinates (NT) and lungs] were collected from the sacrificed mice on days 3 and 6 post-infection for virus titration. On day 3 post-challenge, high virus titers were recovered from both the nasal turbinates and lungs of all mice (Table 3). In contrast, on day 6 post-challenge, no or low virus titers were detected from the nasal turbinates and lungs of some of the adjuvant groups (i.e., ‘vaccine plus EMANON CH-60K’ and ‘vaccine plus hydroxypropyl cellulose’) compared to the vaccine alone group. One of the three mice immunized with alum plus HA vaccine died on day 5 after challenge with viral titers of 6.02 log_10_ PFU/g in its NT and 6.68 log_10_ PFU/g in its lungs. These results demonstrate that none of the adjuvant candidates fully protected the immunized mice from replication of the challenge MA-CA04 virus, but some of the adjuvant candidates, such as EMANON CH-60K and hydroxypropyl cellulose might facilitate the rapid clearance of viruses from mice.

**Table 3:** Virus titers in the respiratory tract of immunized mice after challenge^a^.

	Virus titers (mean log _10_ PFU ± SD/g) in:			
	Nasal turbinates		Lungs	
Immunogen	Day 3 p. i.	Day 6 p. i.	Day 3 p. i.	Day 6 p. i.
PBS	6.4 ± 0.1	5.4, NA^b^, 5.3	6.9 ± 0.0	6.0, NA, 5.7
EMANON CH-25	6.4 ± 0.1	5.3 ± 0.2	7.1 ± 0.1	5.8 ± 0.2
EMANON CH-60K	6.4 ± 0.1	5.5, NA, 6.2	7.1 ± 0.2	5.8, NA, 5.8
Hydroxypropyl cellulose	6.6 ± 0.2	5.9 ± 0.0	7.0 ± 0.1	6.0 ± 0.1
Polyoxyethylene polyoxypropylene glycol	6.8 ± 0.1	5.4 ± 0.2	6.9 ± 0.2	6.0 ± 0.4
Sodium benzoate	6.4 ± 0.1	5.0 ± 0.6	6.7 ± 0.2	6.2 ± 0.2
Sodium sulfite	6.1 ± 0.3	5.5, NA, 5.4	7.0 ± 0.1	5.8, NA, 6.3
Vaccine alone	6.3 ± 0.1	4.3 ± 0.8	6.9 ± 0.1	4.9 ± 0.9
Vaccine + EMANON CH-25	6.2 ± 0.1	3.3 ± 1.0	7.1 ± 0.1	4.0, 4.9, ND
Vaccine + EMANON CH-60K	6.1 ± 0.2	1.7, 3.6, ND^c^	7.1 ± 0.2	ND, 5.1, ND
Vaccine + hydroxypropyl cellulose	6.5 ± 0.1	4.3, ND, ND	7.0 ± 0.2	5.9, ND, ND
Vaccine + polyoxyethylene polyoxypropylene glycol	6.1 ± 0.2	4.1 ± 0.1	7.0 ± 0.1	5.0 ± 0.2
Vaccine + sodium benzoate	6.2 ± 0.0	5.5, 4.3, ND	7.0 ± 0.2	5.1 ± 1.2
Vaccine + sodium sulfite	6.2 ± 0.2	4.1 ± 0.5	7.0 ± 0.1	4.4 ± 0.9
Vaccine + alum	6.5 ± 0.3	3.7, ND, NA	6.9 ± 0.1	3.4, ND, NA

^a^*Six 6-week-old BALB/c mice per group were intramuscularly immunized with the indicated immunogens (100 μl) twice with a 2-week interval between immunizations and then challenged with 10 MLD_50_ of MA-CA04 virus 3 weeks after the second immunization. Nasal turbinates and lungs were collected from mice on days 3 and 6 post-infection (p. i.), and virus titers were determined in MDCK cells by use of plaque assays.*

^b^*NA, not applicable because the mice died on day 5 post-infection.*

^c^*ND, not detectable*.

Adjuvants can improve vaccine efficacy, and the discovery of new adjuvants that are safe and effective is an important step in vaccine development. Here we screened 46 compounds from injectable drug additives approved in Japan, and identified 20 compounds that enhanced the efficacy of the influenza vaccine against lethal virus challenge in a mouse model. These promising adjuvant candidates are of value to the rational design and development of novel and improved adjuvanted vaccines for not only influenza but also other infectious diseases.

Pharmaceutical excipients are used to enhance the action, solubility and/or stability of the active ingredients. In most of cases, excipients themselves are thought to have no medical benefit; however, here we demonstrated that 20 injectable excipients have adjuvanticity, including 15 novel adjuvant candidates. Five of the 20 hit compounds have shown adjuvant effects for other antigens, but their adjuvanticity for influenza vaccine had not been reported until now: dextran 40 (Houston et al., 1976; Joo and Emod, 1988), ethanol (Das et al., 2012), gum arabic (Galliher-Beckley et al., 2015), polyoxyethylene polyoxypropylene glycol (160E.O.) (30P.O.) (Snippe et al., 1981; Shakya and Nandakumar, 2013), and RHEODOL AO-15V (sorbitan sesquioleate) (Fukanoki et al., 2000, 2001). Three of these five compounds (i.e., ethanol, gum arabic, and RHEODOL AO-15V) were used to prepare the nanoemulsion W805EC, the OW-14 emulsion, and a water-in-oil-in-water emulsion, respectively (Fukanoki et al., 2000, 2001; Das et al., 2012; Galliher-Beckley et al., 2015) and showed their adjuvant effects as part of these emulsions; however, the adjuvant effect of each compound *per se* has not previously been shown. To our knowledge, this is the first report to demonstrate their adjuvanticity outside of an emulsion formulation.

Combining adjuvants is one of the most promising approaches to improve their efficacy and safety. GlaxoSmithKline (GSK) Biologicals has been developing various Adjuvant Systems, such as AS01, AS02, AS03, and AS04, in which classical adjuvants are combined with immunomodulators to induce innate and/or adaptive immune responses (Garcon et al., 2007; Didierlaurent et al., 2009; Morel et al., 2011). The 20 compounds identified in this study would be appropriate for combining because they have already been proven safe in humans as injectable excipients. In addition, it would be worth testing vaccine antigen/adjuvant emulsions to see if they could improve immune responses to influenza vaccines. Since some emulsions can act as a depot for the antigen, emulsion adjuvants prepared by using the 20 compounds identified in this study may lead to prolonged and sustained high antibody titers in immunized individuals. These strategies would exploit any synergistic effects of these compounds for the development of adjuvanted human vaccines.

## Author Contributions

HF, MY, TW, and YK designed the experiments. HF performed the experiments. MY selected and purchased all of the chemicals. HF, TdSL, TW, and YK analyzed the data. TW and YK oversaw the study. HF, TdSL, TW, and YK wrote the manuscript. All authors reviewed and approved the manuscript.

## Conflict of Interest Statement

YK has received speakers’ honoraria from Toyama Chemical and Astellas, Inc.; grant support from Chugai Pharmaceuticals, Daiichi Sankyo Pharmaceutical, Toyama Chemical, Tauns Laboratories, Inc., and Otsuka Pharmaceutical, Co., Ltd.; and is a founder of FluGen. The remaining authors declare that the research was conducted in the absence of any commercial or financial relationships that could be construed as a potential conflict of interest.

## Footnotes

1 https://www.cdc.gov/flu/professionals/vaccination/effectiveness-studies.htm

2 www.r-project.org
