# Supplementary material for: Injectable Excipients as Novel Influenza Vaccine Adjuvants
Source: Front Microbiol. 2019 Jan 24;10:19. doi: 10.3389/fmicb.2019.00019 (PMC6353828; doi:10.3389/fmicb.2019.00019)
Supplement: Supplementary file 1 [file Presentation_1.pptx]

## Slide 1
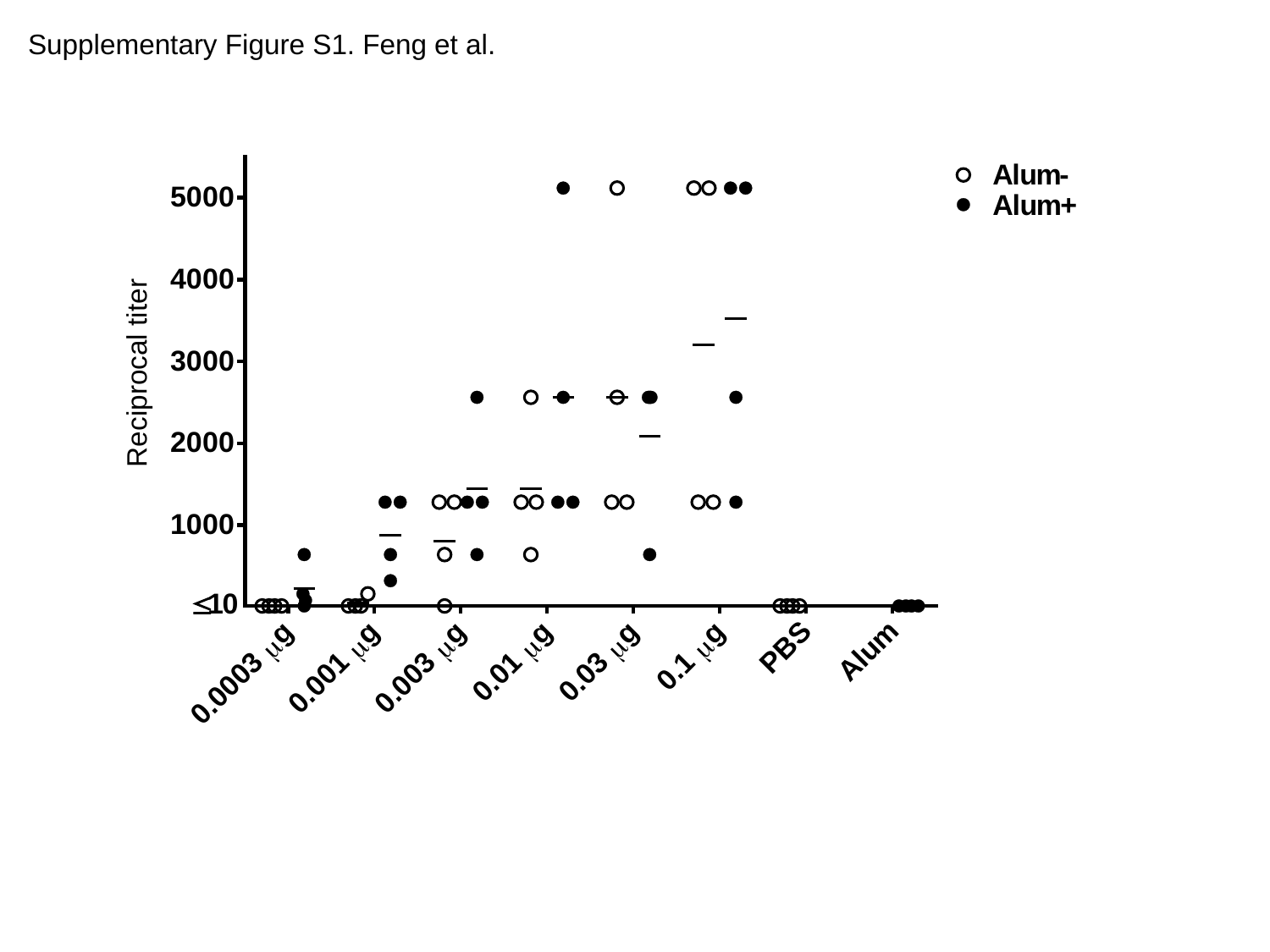

Supplementary Figure S1. Feng et al.
Reciprocal titer

## Slide 2
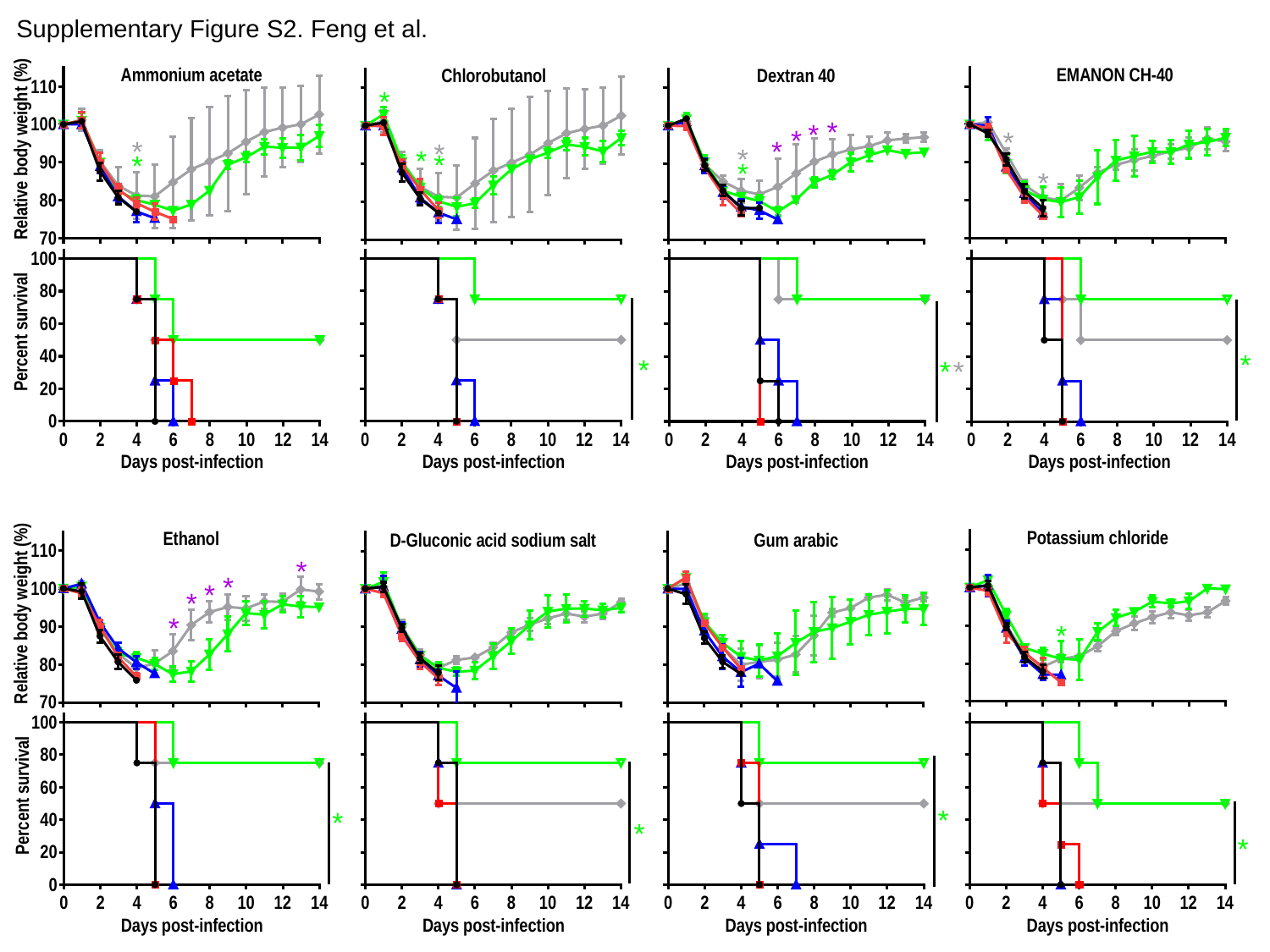

Supplementary Figure S2. Feng et al.

## Slide 3
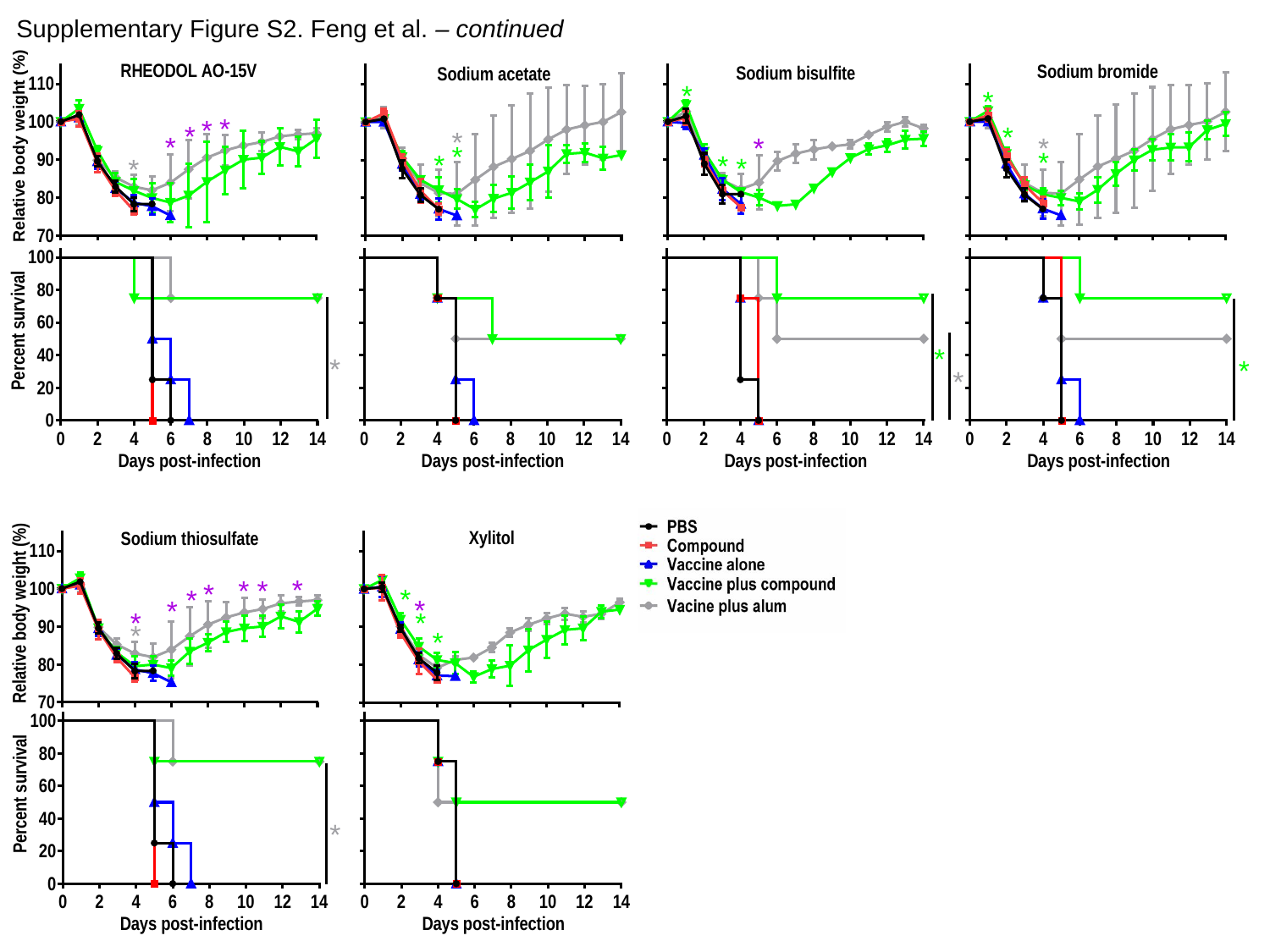

Supplementary Figure S2. Feng et al. – continued
