# Supplementary material for: Injectable Excipients as Novel Influenza Vaccine Adjuvants
Source: Front Microbiol. 2019 Jan 24;10:19. doi: 10.3389/fmicb.2019.00019 (PMC6353828; doi:10.3389/fmicb.2019.00019)
Supplement: Supplementary file 3 [file Data_Sheet_2.doc]

**Supplementary Information**

- Supplementary Figure legends
- Supplementary Table S1. Resources, antibody titers, and protective efficacy of the injectable excipients used in this study (excel file)

**Supplementary Figure legends**

**Supplementary Figure S1. Determination of an appropriate antigen dose for the screen.**

Six-week-old mice were immunized twice with a two-week interval between immunizations. Five doses (0.0003 µg, 0.001 µg, 0.01 µg, 0.03 µg, and 0.1 µg) of HA vaccine with or without alum were examined. Blood was collected two weeks after the boost immunization and antibody titers were measured by use of an ELISA. The lines represent mean antibody titers (n=4).

**Supplementary Figure S2. Body weight changes and survival of immunized mice after lethal challenge.** Six-week-old BALB/c mice were immunized with PBS, compound only, HA vaccine only. or compound-adjuvanted HA vaccine twice with a two-week interval between immunizations. Mice were intranasally challenged with 10 MLD_50_ of MA-CA04 virus three weeks after the second immunization. Body weight and survival were monitored daily for 14 days. The body weight data shown are means and standard deviation (SD; n=4). Green asterisks indicate a significant difference between the vaccine alone and the vaccine plus compound group; Gray asterisks indicate a significant difference between the vaccine alone and the vaccine plus alum group; purple asterisks indicate a significant difference between the vaccine plus alum and the vaccine plus compound groups. * *P* <0.05.
